# Meningococcal disease outbreak related to the World Scout Jamboree in Japan, 2015

**DOI:** 10.5365/WPSAR.2016.7.3.007

**Published:** 2017-05-08

**Authors:** Mizue Kanai, Hajime Kamiya, Alison Smith-Palmer, Hideyuki Takahashi, Yushi Hachisu, Munehisa Fukusumi, Takehito Saitoh, Makoto Ohnishi, Tomimasa Sunagawa, Tamano Matsui, Kazunori Oishi

**Affiliations:** a Field Epidemiology Training Program, National Institute of Infectious Diseases, Tokyo, Japan.; b Division of Global infectious diseases, Department of Infection and Epidemiology, Graduate School of Medicine, Tohoku University, Miyagi, Japan.; c Infectious Disease Surveillance Center, National Institute of Infectious Diseases, Tokyo, Japan.; d Health Protection Scotland, Glasgow, United Kingdom.; e Department of Bacteriology I, National Institute of Infectious Diseases, Tokyo, Japan.; f Department of Epidemiology for Infectious Diseases, Osaka University Graduate School of Medicine, Osaka, Japan.

## Abstract

**Problem:**

Six invasive meningococcal disease cases occurred among Scottish and Swedish nationals associated with the World Scout Jamboree (WSJ), an international mass gathering, held in Japan. The index case developed symptoms while returning home. The strains from all six cases were identical and seldom seen in Japan.

**Context:**

Over 33 000 participants from 155 countries attended WSJ. At the Jamboree site, participants of the North of Scotland’s and Sweden’s units camped within the same subcamp and kept the same schedule of events. No information was available about the Swedish and Scottish cases’ close personal contact history.

**Action:**

Health Protection Scotland investigated Scottish cases, conducted active case finding, provided chemoprophylaxis, vaccinated close contacts and advised Scottish WSJ participants and contacts to seek medical care if they developed symptoms. The Public Health Agency of Sweden recommended chemoprophylaxis to all participants in Sweden. In Japan, the Ministry of Health, Labour and Welfare (MHLW) requested the Scout Association of Japan advise all participants to seek medical attention if they developed symptoms. MHLW shared information about the event with local authorities, medical associations, and the Ministry of Education, Culture, Sports, Science and Technology.

**Outcome:**

No additional case related to WSJ has been reported. This outbreak highlighted the risk for international spread of invasive meningococcal disease at international mass gatherings.

**Discussion:**

Assessing risk, educating participants, enhancing surveillance and sharing timely information among related countries are significant for prevention and response against invasive meningococcal disease outbreaks at mass gatherings.

## Problem

The 23^rd^ World Scout Jamboree (WSJ), held in Yamaguchi prefecture, Japan, from 28 July to 8 August 2015, was a mass gathering in which over 33 000 participants attended from 155 countries. Throughout the event, participants slept in shared tents and participated in socializing activities. (*1*) These types of close interactions can increase the risk of infectious diseases.

Six cases of invasive meningococcal disease (IMD) related to WSJ, including three scouts and one parent from Scotland and two scouts from Sweden, were reported by public health agencies in the United Kingdom and Sweden after WSJ had ended. The index case developed symptoms while returning to Scotland. The strains from all six cases were identical and belonged to serogroup W, (*2*) a serogroup that has seldom been documented in  Japan. The Ministry of Health, Labour and Welfare (MHLW) of Japan was notified and began an investigation. This paper summarizes the experience and lessons learnt from the IMD outbreak in the mass-gathering setting in Japan.

## Context

WSJ is an official event of the World Organization of the Scout Movement that is designed for scouts aged 14 to 17 to live together, experience different cultures and take part in activities. (*1*) After Japan, the United Kingdom and Sweden were represented by the largest number of participants at the 2015 WSJ.

**Fig. 1:**
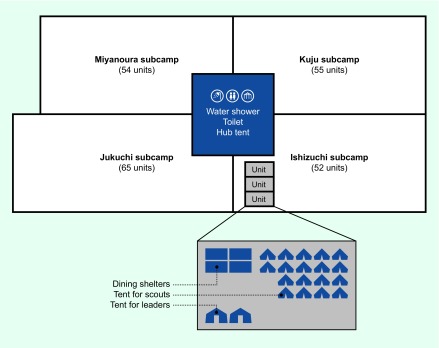
Site map (Western Hub) of World Scout Jamboree 2015

At the Jamboree site, there were three hubs (Northern, Eastern and Western), each with four subcamps. The North of Scotland and Sweden units camped at Ishizuchi subcamp in the Western hub with 50 other units from different parts of the world. Each unit comprised 40 participants (36 scouts and four leaders) with two scouts per tent. Shared kitchen, shower and bathroom facilities were at the centre of each hub (**Fig. 1**). Scouts located in the same subcamp shared one schedule of events during WSJ. There were discotheques and campfire events at night that all participants were expected to attend.

Western Hub had the following subcamps and units: 52 units in Ishizuchi subcamp, 65 units in Jakuchi subcamp, 55 units in Kuju subcamp, and 54 units in Miyanoura subcamp. Each unit consisted of 36 scouts and four leaders. Two scouts shared a tent.

During WSJ, every participant was required to report his or her health condition to WSJ headquarters every morning. Collected information was reported to the local jurisdiction of WSJ every day for syndromic surveillance. No participants developed meningitis symptoms during WSJ.

Between 8 and 19 August 2015, four meningococcal cases were confirmed by Health Protection Scotland (HPS), including three members of the North of Scotland WSJ unit and one family member of a participant. The onset date for the first case was 8 August (during return travel to Scotland), for the second case 11 August, the third 12 August and the fourth 16 August. The fourth case was the secondary case, a household contact (parent) of a scout from the North of Scotland unit. All four cases in Scotland received proper treatment and recovered without complications. (*3*, *4*) A fourth scout, who was not a case, had a sore throat with onset 8 August, was prescribed amoxicillin on 10 August and was diagnosed with Group G streptococcus by throat swab microbiological analysis.

Two confirmed cases from the Swedish WSJ unit were reported by Public Health Agency of Sweden. One case, a scout who returned from Japan on 9 August, developed symptoms on 14 August and recovered after intensive treatment. (*3*, *4*) This case attended a cultural day at the campsite hosted by the North of Scotland unit. The second case, also a scout, developed symptoms on 12 August and was later confirmed by serology. (*4*) No information was available about close personal contact among the cases.

All six cases were confirmed as *Neisseria meningitidis* strain W: P1.5, 2, 36–2: F1–1: ST-11 (cc11) (see **Table 1**), which was indistinguishable from the strain that has been increasing in England since 2009 and a recently increasing IMD capsular group W in Scotland. (*2*, *4*) Based on data available (March 2013 to July 2016), this strain has not been reported recently in Japan. (*5*) No IMD case (a nationally notifiable disease) associated with this outbreak has been reported in Japan as of 5 March 2016.

**Table 1:** Linelist of confirmed cases of meningococcal outbreak associated with World Scout Jamboree (*n* = 6)

Case No.	Unit	Onset date	Symptoms	Serogroup	Outcome
1	North of Scotland Scout	8 Aug	Conjunctivitis, Fever, Headache, Nausea	W (ST11)	Remission
2	North of Scotland Scout	11 Aug	Cough, Headache, Neck stiffness	W (ST11)	Remission
3	North of Scotland Scout	12 Aug	Sour throat, Fever, Headache, Photophobia	W (ST11)	Remission
4	Parent of a North of Scotland scout	16 Aug	Vomit, Myalgia, Headache, Photophobia	W (ST11)	Remission
5	Sweden Scout	14 Aug	Signs of meningitis and septicemia	W (ST11)	Remission
6	Sweden Scout	12 Aug	No info	W (ST11)	Remission

In this outbreak associated with WSJ, the attack rate (AR) among United Kingdom participants (scouts and leaders) was 102.2 cases per 100 000 participants (three cases in 2934 participants). For Swedish participants (scouts and leaders), the AR was 136.4 cases per 100 000 participants (two cases in 1466 participants). Among all participants in Ishizuchi subcamp, the AR was 240.4 per 100 000 (five cases in 2080), and for all WSJ participants (scouts and leaders), the AR was 19.5 per 100 000 (five in 25 649).

## Action

According to HPS, active investigation was conducted in Scotland and chemoprophylaxis and vaccination were appropriately provided for close contacts. (*4*) In addition, HPS e-mailed a letter to all scouts and leaders in the United Kingdom who attended WSJ to alert them to the incident and the signs and symptoms of meningitis and to advise them to seek medical care if they became symptomatic. (*3*, *4*)

The public health actions in Sweden included a recommendation for all participants to seek health care to receive chemoprophylaxis and have nasopharyngeal and throat swabs taken. (*4*)

**Fig. 2:**
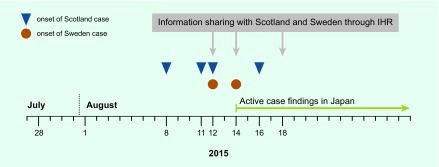
Timeline of the onset of meningococcal cases related with World Scout Jamboree

In Japan, MHLW held a teleconference with Scottish authorities to collect information on the cases. On 14 August, MHLW requested the Scout Association of Japan to advise WSJ participants to visit hospitals as soon as possible if they developed symptoms of meningococcal disease. On 19 August, MHLW advised all local health authorities to inform all medical institutions in their areas of the notice sent from the Scout Association of Japan (**Fig. 2**).

## Outcome

No additional IMD case related to WSJ has been reported. An IMD outbreak occurred across multiple countries and was associated with a mass gathering. The event, WSJ, brought a large number of people together from all over the world, including countries with high incidences of meningococcal disease. This meningococcal outbreak highlighted the potential risk of IMD outbreaks in mass gatherings even in low incidence countries.

## Discussion

Meningococcal disease is listed in the International Health Regulations as a disease with potential serious public health impact and rapid international spread. (*6*) In 2000 and 2001, meningococcal outbreaks caused by serogroup W were reported in England and France, respectively, both related to travellers to the Hajj. (*7*)

The AR reported in the 23rd WSJ (240.4 cases per 100 000 in Ishizuchi subcamp and 19.5 per 100 000 for all WSJ) far exceeded the annual incidence rate in Japan in 2014 (0.03 per 100 000 population), (*5*) which was lower than that for the United States of America (0.3 per 100 000 population in 2009), Europe (0.9 per 100 000 population in 2009) and Australia (1.2 per 100 000 population in 2009). (*8*) One IMD outbreak has been reported in Japan in recent decades. (*9*) The carriage rate of *N. meningitidis* in the nasopharynx has been reported as 0.4% in Japan, (*10*) which is lower than that for other countries.

The risk of transmission of meningococcal disease can increase with close and prolonged contact, such as among household members, or with kissing or sharing food or drinking utensils with patients and carriers. (*3*) Although there was no information available about close personal contact among participants, the close living environment and events of WSJ, such as discotheques and campfires, may have increased the risk of spreading meningococcal disease. Even when the incidence of IMD is low in the host country, a mass gathering produces special circumstances that can lead to IMD outbreaks among participants.

In the United Kingdom, serogroup B has been responsible for the majority of IMD cases, as well as for most European countries over the past decades; (*11*) however, serogroup W has increased rapidly since 2009 and accounted for 25% of all IMD cases in England in 2014 and 2015. (*11*) The strain associated with this outbreak was indistinguishable from the strain that has been circulating in England and Scotland recently.

A total of 77 IMD cases were reported to the Japanese National Epidemiological Surveillance of Infectious Disease system, between 25 March 2013 and 26 July 2015. Among reported cases, four were serogroup W. (*5*, *12*) Genetic analysis of these strains, however, revealed that they were not identical to the WSJ-associated strain. Based on these findings, we speculate that the index case was carrying *N. meningitidis* before attending WSJ or acquired meningococcal from someone who was a carrier at the event.

Until Scottish authorities notified Japan about the case, Japanese authorities were not aware of this outbreak because there were no domestic IMD cases related to WSJ. Once Scottish authorities notified Japan, it was able to begin a risk assessment for Japanese participants and the general public. This situation highlights the importance of international information sharing. Information on epidemiological investigation and gene analysis from other countries is essential to understanding the outbreak and response in a correct and timely manner. (*13*)

In an outbreak, it is recommended that prophylaxis be given to all close contacts of a case and that people identified as high risk be vaccinated. (*3*, *14*, *15*) Tetravalent meningococcal conjugate vaccines against groups A, C, Y and W (MCV4) have been available in Japan since May 2015 and could provide an effective measure to prevent IMD outbreaks at mass gatherings in Japan. Currently, however, MCV4 is not included in the routine vaccination schedule due to low incidence of IMD. MCV4 vaccination of high-risk groups merits serious consideration for protecting against this potentially fatal disease with documented international transmission.

A mass gathering produces special circumstances that can lead to IMD outbreaks among participants even in low incidence countries. Keys to an early and effective response include identifying potential risks, raising awareness among all participants, enhancing surveillance and strengthening communication among participant countries. Prophylaxis is recommended for all close contacts, and vaccination is an available prevention and control measure.
